# Partial Purification of a Megadalton DNA Replication Complex by Free Flow Electrophoresis

**DOI:** 10.1371/journal.pone.0169259

**Published:** 2016-12-30

**Authors:** Caroline M. Li, Yunan Miao, Robert G. Lingeman, Robert J. Hickey, Linda H. Malkas

**Affiliations:** 1 Department of Molecular and Cellular Biology, Beckman Research Institute at City of Hope, Duarte, California, United States of America; 2 Department of Molecular Medicine, Beckman Research Institute at City of Hope, Duarte, California, United States of America; Istituto di Genetica Molecolare, ITALY

## Abstract

We describe a gentle and rapid method to purify the intact multiprotein DNA replication complex using free flow electrophoresis (FFE). In particular, we applied FFE to purify the human cell DNA synthesome, which is a multiprotein complex that is fully competent to carry-out all phases of the DNA replication process in vitro using a plasmid containing the simian virus 40 (SV40) origin of DNA replication and the viral large tumor antigen (T-antigen) protein. The isolated native DNA synthesome can be of use in studying the mechanism by which mammalian DNA replication is carried-out and how anti-cancer drugs disrupt the DNA replication or repair process. Partially purified extracts from HeLa cells were fractionated in a native, liquid based separation by FFE. Dot blot analysis showed co-elution of many proteins identified as part of the DNA synthesome, including proliferating cell nuclear antigen (PCNA), DNA topoisomerase I (topo I), DNA polymerase δ (Pol δ), DNA polymerase ɛ (Pol ɛ), replication protein A (RPA) and replication factor C (RFC). Previously identified DNA synthesome proteins co-eluted with T-antigen dependent and SV40 origin-specific DNA polymerase activity at the same FFE fractions. Native gels show a multiprotein PCNA containing complex migrating with an apparent relative mobility in the megadalton range. When PCNA containing bands were excised from the native gel, mass spectrometric sequencing analysis identified 23 known DNA synthesome associated proteins or protein subunits.

## Introduction

DNA replication is a process that requires the concerted action of numerous proteins and enzymes. We investigated the potential usefulness of free flow electrophoresis (FFE) as a reproducible and gentle technique for purifying and studying the DNA synthesome. The DNA synthesome is a multiprotein DNA synthetic apparatus of mammalian cells, initially termed the multiprotein DNA replication complex or MRC [1, 2], and was used by us to analyze the mechanics of mammalian DNA replication and better understanding the mechanism of action of anticancer drugs that inhibit the DNA replication process [3, 4].

Previous purification methods of the DNA synthesome involved differential centrifugation, salt extraction, glycerol and sucrose gradient centrifugation, and anion exchange chromatography [2, 5–10]. Using these methods, the DNA synthesome from HeLa cells was isolated as a 21S complex following centrifugation through a 10–35% glycerol gradient containing 0.5 M KCl. This 21S complex also exhibited cell-free simian virus 40 (SV40) origin-specific and tumor antigen (T-antigen) dependent DNA replication activity [5]. Proteins previously identified as part of the human DNA synthesome include: DNA polymerases α, δ, and ɛ (Pol α, δ, and ɛ), DNA ligase I, topoisomerase I and II (topo I and II), proliferating cell nuclear antigen (PCNA), replication factor C (RFC), replication protein A (RPA), DNA primase, flap endonuclease 1 (FEN1), poly(ADP-ribose) polymerase (PARP), and DNA methyltransferase (DNMT1) [2, 5–7, 9–13] to sequentially carry-out the biochemical reactions associated with the initiation, elongation and termination phases of the DNA replication process. Each of the identified protein components of the DNA synthesome have specific roles in mammalian DNA replication [14, 15]. In an effort to preserve the overall structure, while maintaining the activity of the replication protein complex, we utilized free flow electrophoresis (FFE) to purify the DNA synthesome.

FFE is an electrophoresis separation method developed over the last 50 years [16]. Under an electrical field, analytes move perpendicular to a flow of electrolytes in the separation chamber, while at the same time being resolved from one another by virtue of their apparent charge differences. Many groups successfully applied this technique to separate various sized components, ranging from intact cells, organelles [17, 18], plasma membrane vesicles [19], and large protein complexes [20] down to small peptides [21], organic compounds, and inorganic compounds [16]. One advantage of using FFE to separate large complexes is that the separation occurs in a liquid phase rather than through gels or a solid phase during column chromatography. Liquid phase separation is ideal as a gentle approach for purifying functionally intact multiprotein complexes because protein complexes have a greater risk to disassemble when moving from a liquid phase into either a molecular sieve network of a gel [20] or onto a solid support matrix used in various types of column chromatography.

We used native interval zone electrophoresis (IZE) to further purify the DNA synthesome. Native IZE is a type of FFE method that employs non-denaturing conditions to resolve complex mixtures of proteins in an electric field across a separation chamber filled with various buffers at different pH values [20]. The native multiprotein complexes or its individual components are resolved from one another according to their apparent ionic charge. The IZE method suppresses sample band broadening which is associated with hydrodynamic flow effects typically observed with the continuous FFE approach [22–24]. To further improve the resolution of individual components of the mixture, proteins pass through a discrete stream of buffer at one pH into an adjacent stream at another pH, thus altering the mobility charge differences of individual molecular species within the mixture. The pH steps also reduce the possibility of proteins precipitating when they reach their isoelectric point [22] because the pH jumps are abrupt and rapid.

We describe the partial purification of the DNA synthesome from HeLa cells by native IZE. Automation of the FFE separation process allowed repeated collection of proteins over multiple cycles. We identified at least two forms of the PCNA protein. Dot blot analysis revealed that only one form was associated with PCNA, topo I, Pol ɛ, Pol δ, RPA and RFC, which are identified protein components of the DNA synthesome. Mass spectrometric protein sequencing revealed additional DNA synthesome proteins. Native IZE separation enhanced purification of the DNA synthesome with a 5.3 fold increase in SV40 origin-specific and T-antigen dependent DNA replication activity. Our results validate the potential benefit of using FFE to rapidly purify a fully active replication competent multi-protein complex from a soluble protein fraction of HeLa cells. Based on native gel separation of the multiprotein complex with mol wt markers, the DNA synthesome complex appears to have a relative molecular weight in the low megadalton (MDa) range.

## Materials and Methods

### Isolation of the DNA Synthesome

HeLa cells were continuously grown in Eagle’s minimum essential medium (Joklik modification with L-glutamine without calcium chloride, Sigma-Aldrich, St. Louis, MO) containing 2 g/L sodium bicarbonate, 5% fetal bovine serum, 5% calf serum, 100 units/mL penicillin, and 0.1 mg/mL streptomycin at pH 7.4 in two 4 L spinner flasks. Cells were grown at 37°C in 5% CO_2_, harvested in mid-log phase (5 x 10^5^ cells/mL), and collected by centrifugation at 250 x g for 5 min. Cell pellets were washed three times by suspending them in ice cold PBS, centrifugation, and removing the supernatant before storing the washed pellet at -80°C.

The DNA synthesome was partially purified to the level of the P4 fraction [5] from a 5 g pellet of HeLa cells with slight modifications. All purification steps were completed at 4°C. Cell pellets were homogenized using a loose-fitting Dounce homogenizer in three pellet vol of 50 mM Tris pH 7.5, 0.25 M sucrose, 5 mM EDTA, 5 mM EGTA, 1 mM PMSF, 1 mM DTT, and 1x protease inhibitor cocktail (Thermo Fisher Scientific, Grand Island, NY). The extract was initially separated by differential centrifugation starting at 570 x g for 10 min to yield a nuclear pellet (the NP fraction) and a supernatant containing the cytosolic extract (the S1 fraction). Mitochondria were pelleted by centrifugation at 10,000 x g for 15 min from the S1 fraction, and the supernatant (the S2 fraction) was further processed to remove microsomes from the S2 fraction by centrifugation at 165,000 x g for 1 h. The supernatant (the S3 fraction) was removed and placed on ice. The NP fraction was suspended in two times the pellet vol of buffer (50 mM Tris, pH 7.5, 1 mM DTT, 0.15 M KCl, 5 mM EDTA, 5 mM EGTA, 1 mM PMSF, and 1x protease inhibitor cocktail), rocked gently for 2 h, and clarified by centrifugation initially at 10,000 x g for 15 minutes to remove larger debris from the NP fraction, followed by centrifugation at 165,000 x g for 1 h (the NE fraction) to prepare a soluble protein fraction. The NE fraction was combined with the S3 fraction, and carefully layered above a sucrose cushion (2 M sucrose, 50 mM Tris, pH 7.5, 0.15 M KCl, 5 mM EDTA, 5 mM EGTA, 1 mM DTT, and 1x protease inhibitor cocktail). After ultracentrifugation using a SW40Ti rotor spun at 40,000 rpm for 17 h using maximum acceleration and no break for deceleration, the lower 20% of the soluble protein above the sucrose cushion was pooled and designated as the P4 fraction.

The P4 fraction was dialyzed into 50 mM Tris-HCl, pH 7.5, 5 mM KCl, 1 mM DTT, 1 mM EDTA, 10% glycerol, 1 mM PMSF, and 1 mM aminoacetonitrile, pH 7.5, using 12–14 kDa molecular weight cut-off (MWCO) SpectraPor 2 dialysis tubing (Spectrum Labs, Houston, TX). Aminoacetonitrile HCl (Thermo Fisher Scientific, Grand Island, NY) was added from a 0.5 M stock concentration adjusted to pH 7.5. After dialysis, the protein retentate was retrieved and stored for later use in aliquots maintained at -80°C.

### PCNA-FLAG Purification

The vector pCMV6-Entry (Origene, Rockville, MD), which contains PCNA with a C-terminal Myc-FLAG (PCNA-FLAG), was stably transfected into MDA-MB-468 breast cancer cells using G418 (1 mg/ml) selection in Dulbecco’s Modified Eagle’s Medium containing 10% fetal bovine serum, 100 units/mL penicillin, and 0.1 mg/mL streptomycin. PCNA-FLAG was affinity purified from cell lysates using anti-FLAG M2 resin and following the directions provided by the manufacturer (Sigma-Aldrich, St. Louis, MO) with appropriate modifications. All steps were performed at 4°C. Cell pellets were lysed by Dounce homogenization using a loose-fitting homogenizer pestle in 50 mM Tris, pH 7.5, 0.25 M sucrose, 5 mM EDTA, 5 mM EGTA, 1 mM PMSF, and 1x protease inhibitor cocktail at 2X the pellet vol. After centrifugation at 1,000 x g for 15 min, the supernatant (the HS fraction) and pellet (the HP fraction) were saved. The HP fraction was suspended in 2 vol of 50 mM Tris, pH 7.5, 0.15 M KCl, 5 mM EDTA, 5 mM EGTA, 1 mM PMSF, and 1x protease inhibitor cocktail and rocked gently for 2 h. The HP fraction was combined with the HS fraction and centrifuged at 10,000 x g for 15 min. The resulting supernatant was clarified at 165,000 x g for 1 h to create a soluble protein fraction containing the FLAG-PCNA protein. Anti-FLAG M2 resin was added to the supernatant, and gently rotated for 1 h in batch format. The resin was loaded into a column, washed with a minimum of 50 column vol of wash buffer (50 mM Tris, pH 7.4, containing 150 mM KCl) and eluted with wash buffer supplemented with 0.1 mg/mL FLAG peptide (Sigma-Aldrich, St. Louis, MO). PCNA-FLAG was dialyzed (50 mM Tris-HCl, pH 7.5, 5 mM KCl, 1 mM DTT, 1 mM EDTA, and 10% glycerol) and frozen at -80°C in aliquots.

### IZE Setup

Spacers (0.2 mm thick), electrode membrane strips, and filter paper strips (0.3 mm thick) were placed between two free flow electrophoresis plates and assembled as described by the manufacturer (FFE Service GmbH, Munich, Germany). The separation chamber temperature was maintained at 10°C. Water was pumped into inlets 1, 4, 6, and 9; while 0.01% sulfanilic acid azochromotrop (SPADNS) was pumped into inlets 2, 3, 5, 7 and 8 to provide visual assurance of an even laminar flow of the liquid phase in the separation chamber. Prior to loading the protein fractions, the separation chamber was coated with 0.2% (hydroxypropyl) methyl cellulose in a solution of 25% glycerol for 1 min at a flow-rate of 360 mL/h, followed by 30 min at a reduced flow-rate of 40 mL/h. The separation chamber was rinsed with water for 10 min at 240 mL/h and then equilibrated in separation buffers for 10 min at 240 mL/h. To establish the pH step gradient across the separation chamber, the buffers pumped through each inlet were as follows: inlet 1 (250 mM mannitol, 100 mM HCl, and 4-(2-hydroxyethyl)morpholine at pH 6.13), inlet 2 and 3 (250 mM mannitol, 20 mM α-hydroxyisobutyric acid and 4-(2-hydroxyethyl) morpholine at pH 6.05), inlet 4 and 5 (250 mM mannitol, 10 mM α-hydroxyisobutyric acid and 4-(2-hydroxyethyl)morpholine at pH 6.6), inlet 6 and 8 (250 mM mannitol, 10 mM α-hydroxyisobutyric acid and 4-(2-hydroxyethyl)morpholine at pH 7.23), inlet 7 (250 mM mannitol, 10 mM α-hydroxyisobutyric acid, 5 mM KCl and 4-(2-hydroxyethyl)morpholine at pH 7.23), and inlet 9 (250 mM mannitol, 150 mM α-hydroxyisobutyric acid, 250 mM Tris base, 50 mM ethanolamine, and 100 mM KOH at pH 9.3). The anode buffer (100 mM HCl and 4-(2-hydroxyethyl) morpholine at pH 6.13) and cathode buffer (100 mM NaOH and 200 mM glycine) were circulated at the electrodes during the entire separation. Mannitol (250 mM) was used as the counter-flow solution.

### Running Samples for IZE

Immediately before IZE purification, the P4 fraction or PCNA-FLAG protein was desalted at 4°C in 5 mM Tris-acetate, pH 7.5, using either a PD SpinTrap G-25 column (GE Healthcare Life Sciences, Pittsburgh, PA) or a 7 kDa MWCO Zeba desalting column (Thermo Fisher Scientific, Grand Island, NY). The relative protein concentration was determined by Bradford assay using BSA as the standard. The P4 fraction was adjusted to a protein concentration between 2.6–12.3 mg/mL before separation.

The voltage, current, power limit, and media pump speed settings were set to 1000 V, 190 mA, 300 W, and 136 mL/h, respectively. Samples (either colored pI marker standard mix (FFE Service GmbH, Germany), the P4 fraction, or PCNA-FLAG protein) were applied to the separation chamber with a sample pump set to 2.1 mL/h with 0.5 mm inner diameter tubing. After 1.5 min, the sample pump was reversed for 1 sec and then stopped. Once the samples reach the separation region of the chamber (15 sec), the voltage was turned on for 10 s and the media pump was adjusted to 36 mL/h. After a 6 min separation, the voltage was turned off, and the samples were collected into a 96-well plate at 236 mL/h over the next 75 sec. Directly from the 96-well plate, the pH of every other well was measured using a modified Freedom EVO robot (Tecan, San Jose, CA) connected to a micro-pH electrode. The optical density of the colored pI marker standard mix was measured at 450 nm using a microplate reader to verify that the separation is routinely reproducible from day-to-day, and that the proper pH steps, timing, and voltage across the separation chamber were maintained throughout the purification.

### Silver Staining

Proteins were resolved by Tris-glycine SDS-PAGE. Five and twelve percent buffered polyacrylamide was used for the 1.5 mm thick stacking and resolving gels, respectively. Selected IZE fractions (0.03 mL) were loaded into each lane. Gels were stained with the Silver Stain Plus kit from Biorad following the manufacturer's directions. Proteins were visualized following silver staining, and their location in the gel compared with the Precision Plus mol wt protein standard (Biorad, Hercules, CA).

### Dot Blot

The nitrocellulose membrane used for the dot-blot analysis was briefly soaked in water followed by 25 mM Tris base, 192 mM glycine, and 20% v/v methanol, pH 8.3. All 96 fractions were passed through a 0.45 μm nitrocellulose membrane by vacuum filtration using the Convertible Filtration Manifold System (Whatman Biometra, Baltimore, MD). For each blot, either 0.1 ml or 0.2 ml of fraction was applied to the membrane. The resulting blot was incubated for 1 h in 2–5% nonfat dried milk before incubation overnight with individual primary antibodies recognizing one of the following proteins: 1:5,000 PCNA (mouse monoclonal, catalog no. SC-56, Santa Cruz Biotechnology, Dallas, TX), 1:500 Topo I (rabbit polyclonal, catalog no. TA307877, Origene, Rockville, MD), 1:500 Pol ɛ subunit 2 (rabbit polyclonal, catalog no. GTX109069, GeneTex, Irvine, CA), 1:500 RPA 32 kDa subunit (mouse monoclonal, catalog no. NA19L, Calbiochem, Darmstadt, Germany), 1:500 Pol δ catalytic subunit (mouse monoclonal, catalog no. 610972, BD Biosciences, San Jose, CA), or 1:500 RFC subunit 4 (rabbit polyclonal, catalog no. SC-20996, Santa Cruz Biotechnology, Dallas, TX). Primary antibodies were then labeled with either 1:5,000 goat anti-mouse IRDye 800CW (catalog no. 926–32210, Li-Cor, Lincoln, NE) for detection on the Odyssey infrared imaging system or 1:10,000 anti-rabbit conjugated with horseradish peroxidase (HRP) (catalog no. 7074S, Cell Signaling Technology, Beverly, MA) for imaging by enhanced chemiluminescence. The signal was quantified using Image Studio Lite (Li-Cor, Lincoln, NE) or Quantity One (Biorad, Hercules, CA) software. The data was normalized on a scale of 0 to 1 by dividing the quantified signal of the fraction with the background subtracted by the maximum quantified signal from the 96 fractions with the background subtracted.

### Blue Native (BN) Gel

IZE fractions were concentrated using a 100 kDa MWCO polyethersulfone ultrafiltration device (Thermo Fisher Scientific, Grand Island, NY) at 4°C, and the final vol of each fraction were adjusted to 0.02 mL with 50 mM Tris, pH 7.5. The protein was loaded into 3–12% gradient NativePAGE Novex 10 well gels (Thermo Fisher Scientific, Grand Island, NY) using the cathode and anode buffers described by the manufacturer, except the buffers were chilled to 4°C. To reduce the potential dissociation of the large complex into component pieces while performing this electrophoresis step, the gels were run at 4°C and 35 V for 18 h using the PowerPac 3000 power supply (Biorad, Hercules, CA). For Coomassie staining, gels were fixed as described by the manufacturer, incubated in Coomassie SimplyBlue Safestain (Thermo Fisher Scientific, Grand Island, NY) for 1 h, and then destained with water.

For Western blotting, proteins were transferred to a PVDF membrane using the Pierce G2 Fast blotter (Thermo Fisher Scientific, Grand Island, NY). The membrane was fixed with 8% acetic acid for 15 min with gentle shaking, rinsed in 100% methanol until the blue color faded, and then briefly rinsed in TBS (20 mM Tris, pH 7.6, and 137 mM NaCl). Ponceau-S solution (Sigma-Aldrich, St. Louis, MO) was added to identify the mol wt marker standards (NativeMark unstained protein standard, Thermo Fisher Scientific, Grand Island, NY), and their relative position on the filter membrane was subsequently marked with a ball point pen. To identify the position of PCNA, the membranes were rinsed with TBS to remove the Ponceau-S solution, blocked for 1 h in 5% milk in TBS containing 0.05% Tween-20 detergent (TBS-T), and incubated with mouse anti-PCNA antibody at the appropriate dilution. The location of the antibody on the membrane was detected using ECL Prime, which reacts with the anti-mouse antibody conjugated with HRP (catalog no. 7076S, Cell Signaling Technology, Beverly, MA.

### SV40 DNA Replication Activity Assay

IZE fractions were concentrated and then desalted into 50 mM Tris, pH 7.5, 5 mM KCl, and 1 mM DTT using 100 kDa MWCO PES concentrators (Thermo Fisher Scientific, Grand Island, NY). Concentrators were prewashed with 0.5 mL of the exchange buffer. Fractions were assayed for T-antigen dependent SV40 DNA replication activity as described previously [5] with modification. The reaction (30 mM HEPES, pH 7.5, 7 mM MgCl_2_, 0.1 mM dATP, 0.1 mM dGTP, 0.1 mM dTTP, 0.1 μM dCTP, 0.2 mM GTP, 0.2 mM UTP, 0.2 mM CTP, 4 mM ATP, 0.5 mM DTT, 1 μCi [α-32P] dCTP, 40 mM phosphocreatine, 2.6 units creatine phosphokinase, 0.25 μg SV40 large T antigen and 50 ng pSVO^+^) was performed at 37°C for 1 h and spotted on either Whatman DE81 ion exchange paper (GE Healthcare Life Sciences, Pittsburgh, PA) or DEAE Filtermat (Perkin Elmer, Waltham, MA) cut to 1 cmx2 cm. After drying, the ion exchange paper was washed twice in 0.1 M sodium pyrophosphate, pH 7.4, twice in 0.3 M ammonium formate, pH 7.4, rinsed in 95% ethanol, and air dried. The activity was measured by scintillation counting. One unit was defined as 1 pmol dNMP incorporated into the plasmid pSVO^+^ DNA after 1 h. The relative protein concentration for each fraction was determined by Bradford assay on a NanoVue Plus microvolume spectrophotometer (GE Healthcare, Pittsburg, PA) at 0.5 mm path length (Biorad, Hercules, CA) using increasing amounts of BSA as the protein standard.

### In-gel Digestion

After separation by IZE and concentration through a 100,000 kDa MWCO membrane, samples were resolved on two blue native (BN) gels loaded with 67% (gel 1) and 33% (gel 2) of the concentrated samples. Gel 1 was stained with Coomassie blue. Western blot analysis using anti-PCNA was performed on gel 2. The Western blot film was then superimposed onto the Coomassie stained gel by mol wt marker alignment, and the identified PCNA containing bands were excised from the native gel. Gel bands were destained three times with 200 μL destaining buffer (40% acetonitrile (ACN) and 200 mM ammonium bicarbonate) for 30 min at 37°C, reduced with 50 μL DTT (15mg/mL) at 80°C for 10 min, and then alkylated in the dark using 50 μL iodoacetamide (18mg/mL) for 45 min at rt. Gel bands were dried for 30 min under vacuum and then rehydrated on ice for 45 min with 10 ng/μL trypsin in 40 mM ammonium bicarbonate and 10% ACN, pH 8. Unabsorbed trypsin solution was discarded, and a 50 μL overlay buffer (40 mM ammonium bicarbonate in 10% ACN) was added to cover the gel to allow trypsin digestion to proceed overnight at 37°C. Peptide fragments within the gel bands were extracted three times with a solution of 50% ACN and 0.1% trifluoroacetic acid. The corresponding peptide solutions were combined, dried completely under vacuum, and stored at -80°C. Prior to LC/MS analysis, samples were dissolved in 10 μL of 1% formic acid.

### Nanoflow LC/MS/MS Analysis

All LC/MS/MS experiments were performed on a Thermo Scientific Orbitrap Fusion Mass Spectrometer equipped with an Easy Spray source and an Easy-nLC1000 system. Mobile phases (MP) were composed of MP-A (0.1% formic acid in water) and MP-B (0.1% formic acid in ACN). Samples (8 μL) were loaded onto a trapping column (Acclaim PepMap100, 75μm x 2cm, C18, 3 μm, 100 Å, Thermo Scientific, Sunnyvale, CA) and separated on an integrated Easy-Spray column emitter packed with PepMap C18, 3 μm, and 100 Å particles (75 μm x 15cm). The column temperature was maintained at 45°C. Samples were desalted for 2.4 min with MP-A at a flow rate of 5 μL/min; the trypsin peptides were separated at a flow rate of 300 nL/min, using a linear gradient from 3% to 35% of MP-B over 8 min. This was followed by a fast gradient to 90% MP-B in 1 min and then 90% MP-B for another min. To improve resolution of the sample components, a longer gradient method was also used that started from 3% to 38% MP-B over 40 min, followed by a fast gradient to 85% MP-B for another min, and ended with 85% MP-B for 4 additional min.

Data was acquired on an Orbitrap Fusion Mass Spectrometer using a resolution of 120,000 (at 200 m/z) for full scans over a 400–1600 m/z range (a maximum of 0.75 s between the full scans), followed by collision induced dissociation fragmentation of the individual peptides (at the top speed setting) and detection of fragmentation ions in the ion trap. Spray voltage was set at 2300 V, S-lens RF level set to 60 and heated capillary set at 275°C. For MS, AGC target was set at 2.5 x 10^5^ and maximum injection time was 100 ms. For the MS/MS experiment, AGC was set to 1x 10^4^ and injection time to 35 ms. Isolation width was set at 2 Da, and collision energy was set to 35%. One microscan was acquired for one spectrum and dynamic exclusion was set at 15 s. XCalibur (3.0.63) software was used for data acquisition. Peptides were identified using three search engines: Proteome Discoverer, the GMP Search, and Mascot. A decoy database was used, and the false discovery rate was set to 5%.

## Results

### Partial Purification using IZE

**Fig 1:**
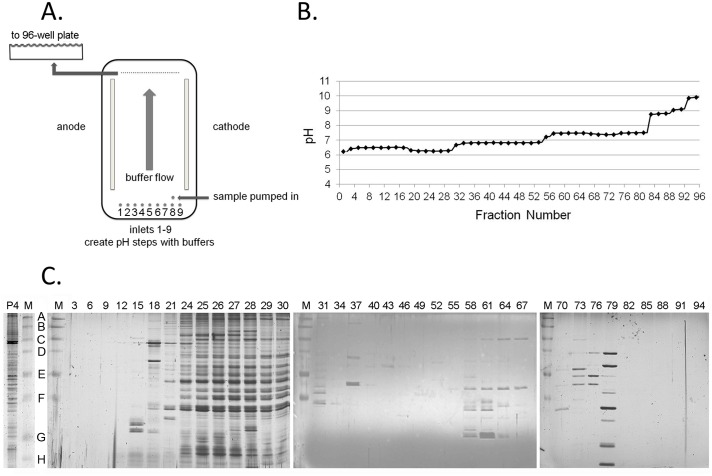
Fractionation of HeLa cell proteins using IZE. (A) Simplified diagram of the FFE separation chamber. (B) pH measured for every other fraction on the 96-well plate after protein separation. (C) Separation of fractions from the IZE by denaturing gel electrophoresis and detection by silver staining. Fraction numbers are labeled on top, and M represents the mol wt marker. P4 represents the protein before the IZE separation. The molecular masses of the markers are 250 kDa, 150 kDa, 100 kDa, 75 kDa, 50 kDa, 37 kDa, 25 kDa, and 20 kDa labeled A to H from top to bottom.

We applied IZE as a technique to increase the purity of the DNA synthesome using its net charge in a mobile liquid based system to resolve it from other proteins and protein complexes found in the soluble protein fraction obtained from mammalian cells. Prior to IZE purification, we verified proper laminar flow, pH steps, voltage, and time conditions for the purification procedure with a dye (SPADNS) and specific pI markers (S1 Fig) as described in the methods section of this manuscript. A series of different buffers were continuously pumped through the individual inlets in order to create discrete laminar flowing streams of buffer that differ in pH from one another when traveling between the anode and cathode (Fig 1A and 1B). Samples were introduced near the cathode side using a peristaltic pump until the sample reached approximately half way up the separation chamber. The sample pump was then reversed for 1 sec to curtail the sample flow before shutting-off the pump. When the samples reach between the anode and cathode with the laminar flow, the electric field was applied for 6 minutes while the flow-rate of the separation buffers was decreased. As samples continue to move through the electrical field perpendicular to the laminar flow, more negatively charged proteins migrate faster toward the anode, and more positively charged proteins move toward the cathode. The heterogeneous mixture of analytes resolve from one another and sharpen as they move towards the anode or cathode and pass through individual streams of separation buffers of differing pH. Together, the individual buffer streams form step-gradients of decreasing pH, each of which sharpens the resolution of the DNA synthesome as it moves toward the anode. After turning off the electric field, the resolved proteins and protein complexes were pumped directly into the wells of a 96-well plate, and the contents of each well were subjected to further analyses in order to determine the location of the DNA synthesome within the buffer stream and to confirm whether the components of the DNA synthesome migrated as an intact and functional protein complex. Because the separation is automated, several purification cycles were sequentially collected and processed for later analysis.

Samples collected from the FFE instrument were visualized after resolution by SDS-PAGE using silver stain (Fig 1C). Fractions 3 and 94 represent samples nearest to the anode and cathode, respectively. Fig 1B shows the pH of every other fraction across the 96 well plate following protein sample collection.

### Optimization of IZE Runs

**Fig 2:**
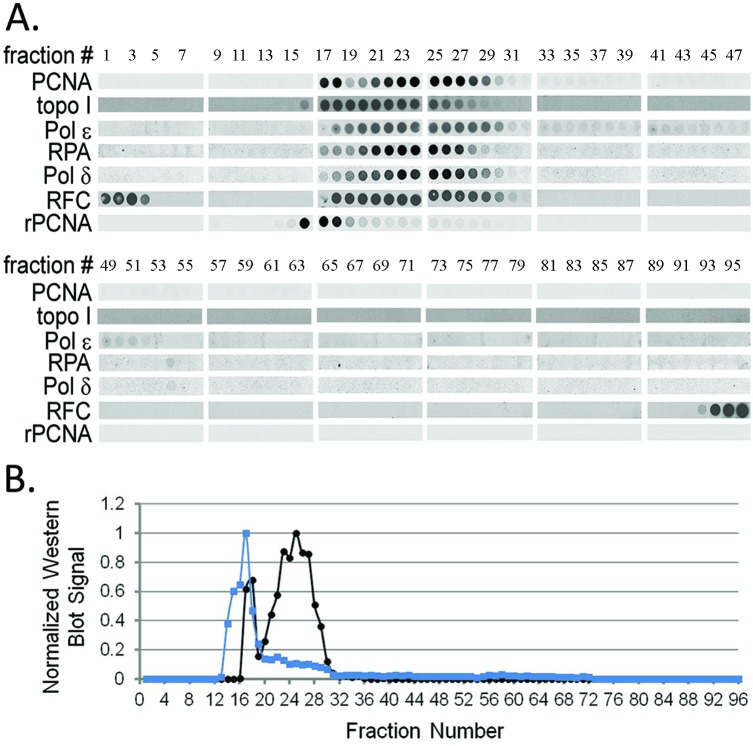
Dot blot analysis of IZE fractions. (A) Dot blot analysis of an IZE separation from the P4 fraction using antibodies that recognize PCNA, Topo I, Pol ɛ subunit 2, RPA subunit 2, Pol δ catalytic subunit, and RFC subunit 4. Recombinant PCNA (rPCNA) was analyzed following IZE separation of PCNA-FLAG expressed and purified protein. Fraction numbers are labeled at the top of the figure. (B) Dot blot quantification of PCNA. The Western blot signal of each fraction for PCNA from the P4 fraction (black circle) and PCNA-FLAG (blue square) was normalized from 0 to 1.

Conditions for the IZE were optimized based on dot blot analysis. For dot blot experiments, all 96 fractions were transferred onto nitrocellulose membranes by vacuum filtration, and specific proteins were detected by Western blot analysis of these filter membranes. Two major peaks were found for PCNA (Fig 2A) from the P4 fraction following IZE separation. The first peak was found at fraction 17, while a second peak was identified at fraction 25. The PCNA-containing peak around fraction 17 from the P4 fraction corresponds to the location where purified recombinant PCNA elutes (Fig 2B). The timing, pump flow rate, and voltage conditions for IZE separation were optimized by monitoring the relative position of colored pI marker standards and the resolution of the two PCNA peaks that were detected by dot blotting. After our optimization studies, we were unable to improve the resolution of the PCNA complex by either altering the resolution time in the separation chamber of the FFE flow cell or by adjusting the voltage used to resolve PCNA and the other DNA synthesome components. In addition, the resolution did not improve by widening the pH step gradient from pH 5.4 to 7.0 (data not shown).

The reproducibility of independent IZE runs performed on six different days was monitored by dot blot analysis with antibody against PCNA. The runs were performed either manually or by robotic automation. The first PCNA peak appeared in fractions 17 or 18. The second PCNA peak appeared in fraction 26 +/- 2, which represents +/- 2 out of 96 fractions or an error of approximately +/- 2.1% and a fraction range equivalent to 4 out of 96 fractions or 4.2%. Differences in the setup or minor changes in the pH or conductivity of the buffers can give rise to these minor variations in electrophoretic mobility. Analysis of the proteins resolved during the first and last purification cycle performed on the same day showed that PCNA peaks consistently in the same fraction.

In an attempt to recover higher protein yields, we loaded increasing amounts of the P4 fraction into the separation chamber and tested the resolution of proteins within the P4 fraction using dot blot analysis with anti-PCNA antibody. Our initial IZE separations injected 0.1 mg of partially purified protein from the P4 fraction (described in the methods section) for each purification cycle. Using dot blot analysis for determining the location of PCNA within the fractions eluting from the separation, we showed that PCNA consistently eluted in the same fractions, and thus the resolving power of IZE was unaffected by increasing the amount of protein loaded into the separation chamber over the six fold range we used in this study. We only checked the resolution when loading up to 0.6 mg of protein.

### Known DNA Synthesome Containing Proteins Co-elute with One Another

We used dot blot analysis to show the elution position of proteins previously identified as part of the DNA synthesome. Dot blot analysis showed that known DNA synthesome proteins, including PCNA, Topo I, Pol ɛ, Pol δ, RPA, and RFC (Fig 2A), co-elute with one another after IZE separation. The results suggest that the DNA synthesome remained intact after partial purification by IZE. Other proteins, most notably PCNA and RFC subunit 4, were found to be complexed or uncomplexed with known DNA synthesome proteins. Replicate dot blot analysis performed on an independent IZE purification run showed equivalent separations of these proteins. To show that the dot blot signal is from the protein of interest and not from a nonspecific signal associated with the primary antibody, denaturing SDS PAGE Western blot analysis showed that the major band is the apparent mol wt for that protein (S2 Fig).

We performed control experiments to determine whether secondary antibodies were falsely detecting proteins being examined in the dot blot analysis. Instead of incubating the nitrocellulose membrane with primary antibody, only the secondary antibody was added. We observed that when the secondary antibody was linked with the IRDye 700, which is a near-infrared fluorescence dye, false signals were detected when scanned at 700 nm; however, when IRDye 800 or HRP was conjugated to the secondary antibody, no false signals from the nitrocellulose membrane were detected. We report here dot blot results with the secondary antibody conjugated with either IRDye 800 or HRP in Fig 2.

### BN PAGE Resolves a MDa PCNA-containing Multi-Protein Complex

**Fig 3:**
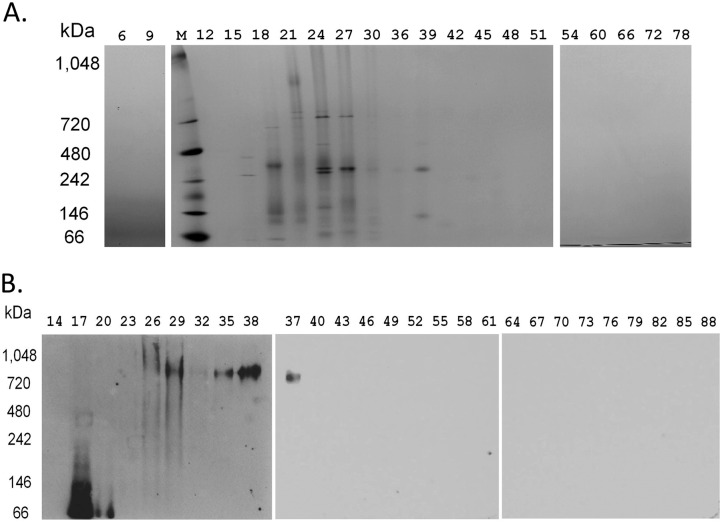
Separation of IZE fractions by BN gel electrophoresis. (A) The gels were stained with Coomassie. (B) Separate gels were transferred to PVDF membrane and probed with PCNA antibody. Fraction numbers are labeled on top of the figure.

To visualize native protein complexes following fractionation of the partially purified cell lysates by IZE, we used Blue Native (BN) gel electrophoresis. We reasoned that BN gels at pH of 7.5 may help protein complexes remain intact, and that they are well suited for resolving MDa sized protein complexes. A feature of BN PAGE is the presence of negatively charged Coomassie G-250, which binds to proteins and migrates with them towards the anode. As proteins move from larger to smaller pores in the gradient polyacrylamide gel, they almost stop migrating when they reach a particular pore size because separation is based on size rather than charge/mass ratio [25]. Since Coomassie G-250 binds to surface hydrophobic regions and reduces protein aggregation, BN gels are often used to resolve membrane proteins in the presence of detergents [26, 27]. To further reduce dissociation of multiprotein complexes, the BN gel is run at low temperature (4°C) and low voltage (35 V) for 18 h [20]. Highly abundant proteins retained the Coomassie stain after electrophoresis, while lower abundant proteins could be detected after additional Coomassie staining (Fig 3A).

To estimate the size of the PCNA associated multiprotein complex, we performed Western blotting with the BN gel and probed with an antibody against PCNA. Native gel mol wt marker proteins were used to provide a relative measure of the mass at any given distance from the wells of the gel. Because of the association of PCNA within the DNA replication apparatus of the cell and the co-migration of PCNA with other proteins having a role in the DNA replication process, it became important to establish whether this PCNA containing multiprotein complex could be the functional DNA synthesome that we had previously reported. It is also important to note that PCNA serves as a processivity factor, which is an essential component of the DNA synthetic process [14] carried-out by the in vitro SV40 DNA replication model system used in the analysis of the mammalian cell DNA synthetic process [28, 29]. The native gel showed two peaks of PCNA with a relative electrophoretic mobility between 800–1,000 kDa (Fig 3B, S3 Fig), at fractions 29 and 38, and a lower mol wt PCNA species which appeared at fraction 17.

### Partial IZE Purification Increases the Specific Activity of T-antigen Dependent SV40 DNA Replication

To determine whether the multiprotein PCNA associated complex eluting from the FFE chamber contained enzymatically active fractions capable of supporting the DNA replication process, we measured the ability of the complex to support in vitro SV40 DNA replication activity. To do so, we assayed every third fraction collected from the IZE separation chamber for its ability to support initiation of DNA replication, DNA unwinding, short RNA oligomer synthesis, and elongation of the DNA template. The plasmid DNA template used in the assay contains the core sequences of the SV40 replication origin [30]. Addition of the SV40 viral large T-antigen to the replication assay mixture containing the multiprotein PCNA associated protein complex results in incorporation of the radiolabeled nucleotide in a T-antigen dependent manner, showing that the multiprotein complex is fully competent to support in vitro SV40 origin and T-antigen dependent DNA replication [3].

**Fig 4:**
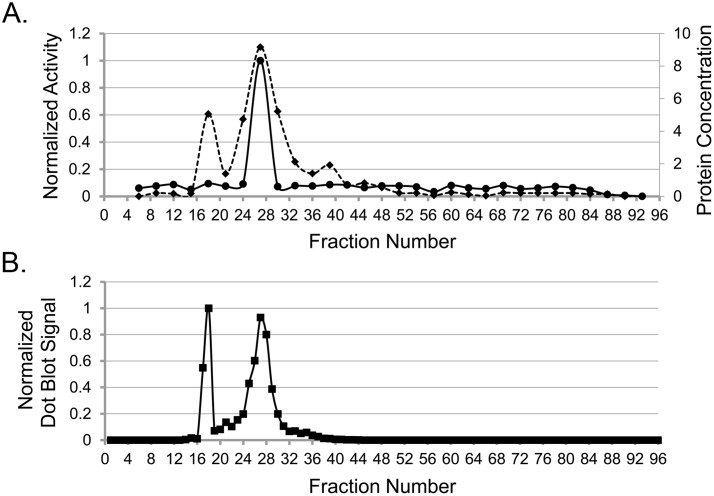
In vitro SV40 DNA replication activity relative to the position of PCNA. (A) Circles with the solid line represent the normalized SV40 DNA replication activity labeled on the left axis. Diamonds with the dotted line represent the protein concentration in μg labeled on the right axis. (B) Squares represent dot blot quantification of PCNA levels from IZE fractions run on the same day. The data were normalized between 0–1.

To measure the DNA replication activity of the purified PCNA associated DNA synthetic protein complex, the PCNA containing fractions from 8 cycles were collected and concentrated through a 100,000 kDa MWCO membrane. This step served as an additional purification step because smaller proteins and protein complexes can be separated from the larger complexes. Importantly, the SV40 DNA replication activity of these IZE fractions was not lost after concentrating the protein through the 100,000 kDa MWCO membrane filter (data not shown), indicating that the proteins needed to support in vitro SV40 origin dependent DNA replication activity are associated with one another in a highly organized and functionally active large complex. Fig 4A shows one major peak for SV40 DNA replication activity at fraction 27. As shown in Fig 4B from Western blot analysis, PCNA was also found within the fractions exhibiting DNA replication activity. The specific activity of the peak for T-antigen dependent DNA replication activity was 11.26+/-0.22 units/mg. One unit is 1 pmol dNMP synthesized into DNA after 1 hour. The specific activity of the protein before IZE separation (the P4 fraction) was 2.11+/-0.06 units/mg. From our assay results, we estimated that the SV40 origin dependent in vitro DNA replication activity of IZE fraction 27 was enhanced 5.3 times over that of the activity of the P4 fraction before IZE separation.

### The MDa Complex Contains Known DNA Synthesome Proteins

To verify the results of the Western blot analysis from IZE fractions, we used mass spectrometry based protein sequencing to validate whether the native gel slices containing PCNA, having a relative electrophoretic mobility between 800–1000 kDa, is the replication competent DNA synthesome. We specifically identified 23 proteins or protein subunits previously reported as being required for the origin and T-antigen dependent DNA replication process; many of whom were identified as components of the DNA synthesome [2, 5–7, 9–13]. Table 1 lists the identified proteins. The roles of these proteins in mammalian DNA replication are described by Smith *et al*. [14].

**Table 1:** Previously identified DNA synthesome proteins or protein subunits identified by mass spectrometry from BN gel with a relative electrophoretic mobility of 0.8–1 MDa.

DNA Synthesome Protein^a^	Protein Symbol
PCNA	PCNA_HUMAN
RFC subunit 2	RFC2_HUMAN
RFC subunit 3	RFC3_HUMAN
RFC subunit 4	RFC4_HUMAN
RFC subunit 5	RFC5_HUMAN
RPA 70 kDa DNA-binding subunit	RFA1_HUMAN
RPA 32 kDa DNA-binding subunit	RFA2_HUMAN
RPA 14 kDa DNA-binding subunit	RFA3_HUMAN
DNA primase small subunit	PRI1_HUMAN
DNA primase large subunit	PRI2_HUMAN
topo III alpha	TOP3A_HUMAN
topo II beta	TOP2B_HUMAN
Pol α catalytic subunit	DPOLA_HUMAN
Pol α subunit B	DPOA2_HUMAN
Pol δ catalytic subunit	DPOD1_HUMAN
Pol δ subunit 2	DPOD2_HUMAN
Pol δ subunit 3	DPOD3_HUMAN
Pol ɛ subunit 2	DPOE2_HUMAN
DNA ligase I	DNLI1_HUMAN
ribonuclease H2 subunit A	RNH2A_HUMAN
DNA (cytosine-5)-methyltransferase 1	DNMT1_HUMAN
FEN1	FEN1_HUMAN
PARP 1	PARP1_HUMAN

^a^Scaffold (Proteome Software) was used to validate MS/MS based peptide and protein identifications. Peptide identifications were accepted if they are greater than 90.0% probability by the Scaffold Local FDR algorithm. Protein identifications were accepted if they achieve an FDR less than 5.0% and contained at least 1 identified peptide. Protein probabilities were assigned by the Protein Prophet algorithm [31].

## Discussion

The native IZE method has advantages and disadvantages over conventional purification methods. A comparison between native IZE with ion exchange chromatography is reasonable since both methods purify molecules or complexes of molecules by their net charge. IZE separation of the P4 fraction followed by concentrating protein fractions through a 100,000 kDa MWCO membrane increased the specific activity of a fraction supporting the in vitro SV40 DNA replication assay by 5.3 times. In comparison, the specific activity of the in vitro SV40 DNA replication assay from P4 increased 2.1 fold after anion exchange chromatography over a 5 ml column [12]. Certainly, the activity enrichment following purification will vary depending on factors such as the time elapsed from first lysing the cells until the purified sample is stored in the freezer, the biophysical properties of the matrix used to dissociate the complex into its components, the number of freeze/thaw cycles, the storage buffer conditions, and the storage temperature.

The primary advantage of using the native IZE method for purifying proteins is that the sample passes through the instrument quickly and gently. For each protein resolution cycle of the IZE method described here, the sample took 2 min to load into the separation chamber, 6 min to separate in the electrical field, and 3 min to move from the separation chamber to the 96 well plate. For 8 cycles, it would take 88 min. As the samples elute from the instrument, they are concentrated through a 100,000 kDa MWCO membrane, while the next cycle runs. In contrast, traditional column chromatography would take much longer, depending on the column size and chromatography media flow rate. We have observed (unpublished) that when the solvent flow rate exceeds 0.1 ml/min in anion exchange chromatography, known components of the DNA synthesome required for origin dependent DNA replication elute in a dispersed manner from the column rather than with the other proteins participating in the DNA replication process, leading to a loss in activity. We surmised that higher flow rates create sheer forces that disrupt complex integrity and the loss in activity may originate from specific components of the large protein complex (the DNA synthesome) dissociating as the complex migrates from the liquid phase onto the chromatography resin. Native IZE is advantageous as a gel-free separation technique because it (1) reduces the type and amount of physical force(s) that can potentially destabilize and, ultimately, break-up protein complexes [20] and (2) reduces the loss of post-translational modifications. It would take 850 min or 14 h to load a 5 ml ion exchange column at 0.1 ml/min with 5 ml of sample diluted in loading buffer, wash the column at 1 ml/min with 10 column volumes of loading buffer, elute the sample with 10 volumes of salt gradient at 0.1 ml/min, and then run 5 volumes of high salt buffer. While there is time in preparing both the chromatography column and the IZE apparatus prior to sample application, the actual sample processing time is substantially shorter through the IZE apparatus than the processing time through the chromatography column.

While not as much protein can be loaded at one time with our IZE protocol, we have automated the procedure so that the DNA synthesome can be resolved from 6 mg of P4 protein in 110 min. For this study, we did not test whether more than 0.6 mg of protein could have been loaded during each cycle and still achieve the same protein resolution. In contrast to the IZE approach, 30 mg of the P4 fraction was loaded onto a 5 ml anion exchange column and processed as noted above [12]. Thus, more protein can be loaded at one time by ion exchange chromatography, but the actual sample processing time on the column is substantially longer than the time for an individual sample to run through the IZE unit.

If high protein yields are important, anion exchange chromatography has its advantages over the IZE method; however, if smaller protein yields are fine with possibly higher specific activity, the IZE method should be considered as a purification step. These considerations are especially important for studying large multiprotein complexes and studying post-translational modifications of proteins. Since the IZE method is gentle and rapid, it can help preserve protein-protein interactions and the post-translational modification status of the protein complex and its individual components during purification. These properties of the IZE method can be especially useful as a rapid approach for facilitating subsequent structure and activity analyses of the complex.

To estimate the size of the active protein complex, the fractions containing replication activity were resolved by BN gel and compared with native mol wt markers. Since native gels separate based on size, shape, and charge, comparing the mobility of native gel markers can only provide an approximate size. For example, proteins that have a compact structure or acidic pI may move through the gradient polyacrylamide gel faster than unfolded proteins or proteins with a very basic pI. In addition, Coomassie G-250 may bind differently to proteins [26], thus influencing the migration of proteins toward the anode. In contrast to traditional native polyacrylamide gels, BN gels mainly separate proteins based on their size rather than their charge/mass ratios. When proteins reach their pore-size limit in a gradient gel, the proteins essentially stop migrating through the gel [25]. In our study following separation by IZE, large multiprotein complexes containing PCNA appear on the BN native gel. The largest PCNA complex has a relative electrophoretic mobility of 0.8–1 MDa compared to the native mol wt marker used in this study (Fig 3B).

It is not clear why dot blot analyses containing fraction 38 and those adjacent to it (Fig 2) did not detect PCNA even though PCNA was detected by Western blot analysis from BN PAGE around fraction 38 (Fig 3B). This observation was reproduced in independent IZE runs performed on three different days. Our observation could be due to a number of different scenarios. One possibility is that the vacuum transfer of proteins in dot blots results in poor complex binding to the nitrocellulose membrane as compared to the electrotransfer of proteins from the native gel to a PVDF membrane. Another possibility is that antibodies may not recognize epitopes buried in proteins, as observed previously [7], after being vacuum filtered onto nitrocellulose membrane. In contrast to the dot blots, proteins from the BN gel are electrotransferred on PVDF membranes and treated in 8% acetic acid and 100% methanol before continuing with Western blot analysis. This may have denatured the complex and exposed epitopes that were not available on the nitrocellulose membrane. It is important to note that fraction 38 also lacked SV40 DNA replication activity (Fig 4A), suggesting that even though the two PCNA complexes have similar size on a native gel, they have different properties. Mass spectrometric analysis of PCNA containing proteins within fraction 38 identified a complex containing PCNA, but failed to identify the other proteins listed in Table 1 with the exception of TOP2B. The most abundant proteins found included epiplakin 1 and fatty acid synthase. While additional studies would be needed to identify the different PCNA containing multiprotein complexes, we know from these studies that the net charges of the replication competent and replication deficient complexes differ, and that the IZE methodology is sensitive enough to resolve these various forms of PCNA-associated complexes.

Early studies described eukaryotic DNA replication as an ordered process involving the activity of many proteins that bind as needed for a particular step but dissociate from the complex when no longer needed [14, 15]. This simplified model was based on in vitro experiments with reconstituted proteins [32]; it was useful at identifying essential proteins and revealing steps in the DNA replication process. However, DNA replication in a cell is more complex involving many more proteins and pathways. For example, many proteins involved in DNA replication undergo post-translational modifications. In addition, kinases play an essential role in regulating the DNA replication and repair process through the cell cycle [33–36].

Contrary to the in vitro assembly model that replication factors are loaded and unloaded sequentially at various points through the cell cycle, our results support the idea that a highly organized multiprotein complex participates in the mammalian DNA replication process because the replication competent core complex remains functionally intact throughout the purification. Previous experiments from our laboratory show that the murine DNA synthesome stays together after treatment with salts, detergents, RNase, and DNase, suggesting that the complex is held together through highly organized and specific protein-protein interactions instead of nonspecific interactions between non-essential cellular macromolecules [1]. Nearly three decades ago, an assembly of replication competent multiprotein complexes was proposed to combine with one another as larger complexes along nuclear matrix filaments to make up replication factories [37] during the S-phase of the cell cycle [38]. In these studies, DNA polymerase α-primase could be purified from regenerating rat liver in several forms, that varied in size as either a 10S or 17S complex or as larger multiprotein complexes of 100S and 150S [38]. In our hands, the replication competent DNA synthesome complex from murine mammary carcinoma cell lines purified with a sedimentation coefficient of 17S [1]. This observation is consistent with the idea that multiple DNA synthesomes could assemble to form replication factories in the nuclei of dividing cells. Replication factories appear as electron-dense bodies in the electron microscope and were found to have proteins that contribute to the DNA replication process [39–41]. The mechanics of these replication factories in the cell remains unknown to this day. Many observations suggest that replication factories stay fixed on a nucleoskeleton while replicating DNA moves through the complex [39, 42–44] rather than the complex moving along the DNA strand.

Considering that DNA replication must occur both rapidly and accurately to copy the cell's genetic material, it would be reasonable that the process involves a complex of proteins ready for DNA synthesis. Diffusion events from sequential loading and unloading of components would considerably slow down the process. A multiprotein complex could allow quick transfer of reaction intermediates from one enzyme to the next. In addition, specific proteins in the complex would be ready for activation at the appropriate time (i.e. throughout late G1 through S-phase). DNA replication and repair proteins may be activated by distinct kinases within the assembled complex instead of transported to where their activity is needed on the nascent DNA strand.

Many essential biological processes within the cell are thought to involve dynamic multiprotein complexes that could quickly channel substrates between enzyme active sites. Examples include protein biosynthesis, RNA transcription, TCA cycle, glycolysis, nucleotide biosynthesis, and even DNA biosynthesis [45–50]. In some cases, protein complexes have been observed in vivo, yet they have been difficult to isolate in vitro possibly due to weak interactions that dissociate during purification [51–53]. If so, purification using gentle methods, such as native IZE, may help isolate large protein complexes; however, if the protein interactions are transient, the complexes would be difficult to isolate.

Several lines of evidence support that we isolated an active DNA synthesome. First, dot blot analysis showed separation of known components of the DNA synthesome either complexed or uncomplexed with PCNA (Fig 2). Complexed PCNA co-eluted with previously identified DNA synthesome containing proteins such as Topo I, Pol ɛ, RPA, Pol δ, and RFC. Uncomplexed PCNA was identified in fractions where immuno-purified PCNA-FLAG eluted following IZE. Second, IZE partial purification followed by protein fraction concentration using centrifugal ultrafiltration enhanced the activity and purity of the DNA synthesome because the specific activity of the SV40 replication origin-specific and T-antigen dependent DNA replication activity increased 5.3 fold. This activity peak was in the PCNA containing fraction (Fig 4) complexed with known DNA synthesome proteins (Fig 2), suggesting that the entire complex is needed for this activity. Third, the fraction with the activity peak also show PCNA eluting from BN gel electrophoresis at 800–1,000 kDa (Fig 3B), which is much larger than uncomplexed PCNA. Fourth, mass spectrometry verified that previously identified DNA synthesome proteins or protein subunits were part of the higher mol wt PCNA complex (Table 1). DNA replication related proteins found in the PCNA multiprotein complex fractions, and the observation of SV40 DNA replication activity in those fractions, support the concept that FFE is a reliable method to rapidly purify the DNA synthesome as a replication competent MDa protein complex.

## Conclusion

In this study, we describe a new method to purify the DNA synthesome involving IZE. We provide evidence that the DNA synthesome isolated as a megadalton (MDa) complex containing the components necessary to support DNA replication. After the IZE and centrifugal ultrafiltration steps, the specific activity of the DNA synthesome increased by 5.3 fold. Other proteins that copurify with the DNA synthesome were identified by mass spectrometry and need to be further validated to understand its role in DNA replication. The IZE method that we describe can be applied to rapidly and gently purify other large multi-protein complexes for biochemical and mass spectrometric analysis.

## Supporting Information

S1 Fig Quality controls of IZE setup before protein was loaded. (A) To ensure even laminar flow in the separation chamber, 0.01% SPADNS was pumped through the inlet tubing lines 2,3,5,7 and 8 prior to addition of samples to the electrophoretic chamber. Water was pumped through the other lines. Black bars show the SPADN absorbance at 450 nm across all 96 fractions. (B) Visible pI markers were detected by absorbance at 450 nm in specific wells of the 96-well plate and displayed as black bars (left axis). The black square represents the measured pH for every other well of the 96 well plate (right axis).
(PDF)

S2 Fig Antibody specificity for the dot blot analysis shown in Fig 2 by SDS-PAGE separation and Western blot analysis. The antibodies recognize (A) PCNA (29 kDa), (B) DNA topoisomerase 1 (91 kDa), (C) DNA polymerase ɛ (60 kDa subunit 2), (D) replication protein A (32 kDa subunit 2), (E) DNA polymerase δ (124 kDa catalytic subunit), or (F) replication factor C (37 kDa subunit 4) from unpurified whole cell extracts.
(PDF)

S3 Fig Native molecular weight marker for Fig 3B. (A) Western blot with antibody recognizing PCNA from fractions off the free flow electrophoresis. The fraction numbers are labeled on top of the corresponding lanes. (B) Coomassie stain of the blue native gel. (C) Western blot of fractions 37–61 from free flow electrophoresis fractions probed with antibody recognizing PCNA.
(PDF)

## Abbreviations

ACN: acetonitrile
BN: blue native
DNMT1: DNA methyltransferase
FEN1: flap endonuclease 1
FFE: free flow electrophoresis
HRP: horseradish peroxidase
IZE: interval zone electrophoresis
MDa: megadalton
MWCO: molecular weight cut-off
PARP: poly(ADP-ribose) polymerase
PCNA: proliferating cell nuclear antigen
Pol α: DNA polymerase α
Pol δ: DNA polymerase δ
Pol ε: DNA polymerase ε
RFC: replication factor C
RPA: replication protein A
SPADNS: sulfanilic acid azochromotrop
SV40: simian virus 40
T-antigen: tumor antigen
topo I: DNA topoisomerase I
topo II: DNA topoisomerase II
