# Supplementary material for: Partial Purification of a Megadalton DNA Replication Complex by Free Flow Electrophoresis
Source: PLoS One. 2016 Dec 30;11(12):e0169259. doi: 10.1371/journal.pone.0169259 (PMC5201288; doi:10.1371/journal.pone.0169259)
Supplement: S1 Fig — (A) To ensure even laminar flow in the separation chamber, 0.01% SPADNS was pumped through the inlet tubing lines 2,3,5,7 and 8 prior to addition of samples to the electrophoretic chamber. Water was pumped through the other lines. Black bars show the SPADN absorbance at 450 nm across all 96 fractions. (B) Visible pI markers were detected by absorbance at 450 nm in specific wells of the 96-well plate and displayed as black bars (left axis). The black square represents the measured pH for every other well of the 96 well plate (right axis). (PDF) [file pone.0169259.s001.pdf]

A.

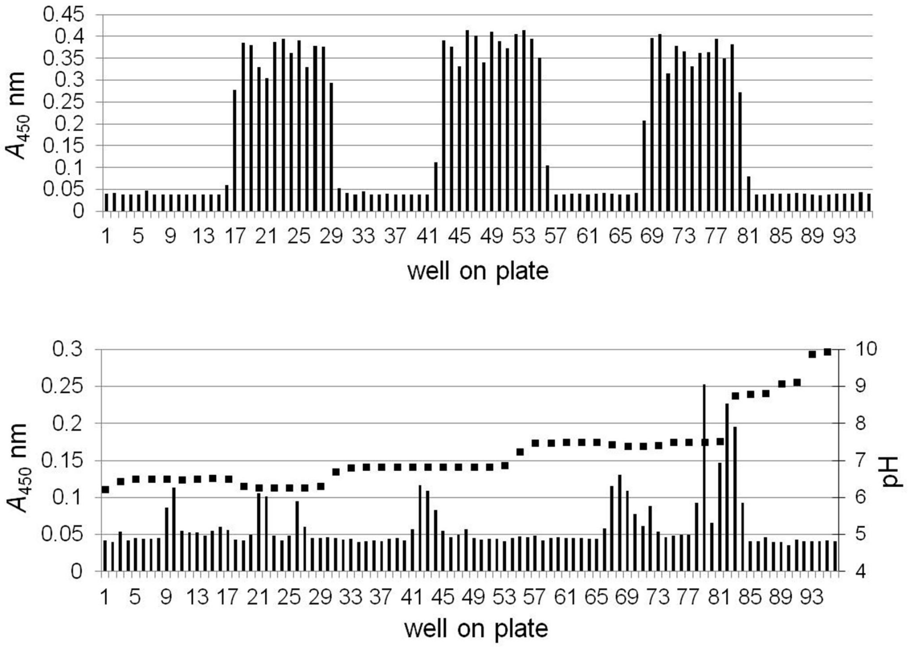


**S1 Fig. Quality controls of IZE setup before protein was loaded.** (A) To ensure even laminar flow in the separation chamber, 0.01% SPADNS was pumped through the inlet tubing lines 2,3,5,7 and 8 prior to addition of samples to the electrophoretic chamber. Water was pumped through the other lines. Black bars show the SPADN absorbance at 450 nm across all 96 fractions. (B) Visible pI markers were detected by absorbance at 450 nm in specific wells of the 96-well plate and displayed as black bars (left axis). The black square represents the measured pH for every other well of the 96 well plate (right axis).
