# Supplementary material for: Partial Purification of a Megadalton DNA Replication Complex by Free Flow Electrophoresis
Source: PLoS One. 2016 Dec 30;11(12):e0169259. doi: 10.1371/journal.pone.0169259 (PMC5201288; doi:10.1371/journal.pone.0169259)
Supplement: S2 Fig — The antibodies recognize (A) PCNA (29 kDa), (B) DNA topoisomerase 1 (91 kDa), (C) DNA polymerase ɛ (60 kDa subunit 2), (D) replication protein A (32 kDa subunit 2), (E) DNA polymerase δ (124 kDa catalytic subunit), or (F) replication factor C (37 kDa subunit 4) from unpurified whole cell extracts. (PDF) [file pone.0169259.s002.pdf]

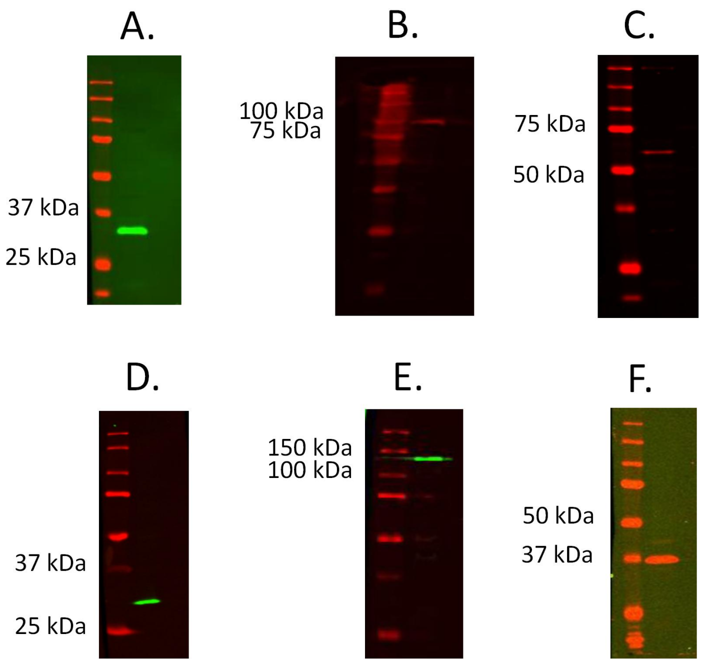

S2 Fig. Antibody specificity for the dot blot analysis shown in Figure 2 by SDS-PAGE separation and Western blot analysis. The antibodies recognize (A) PCNA (29 kDa), (B) DNA topoisomerase 1 (91 kDa), (C) DNA polymerase ε (60 kDa subunit 2), (D) replication protein A (32 kDa subunit 2), (E) DNA polymerase δ (124 kDa catalytic subunit), or (F) replication factor C (37 kDa subunit 4) from unpurified whole cell extracts.
