# Supplementary material for: Partial Purification of a Megadalton DNA Replication Complex by Free Flow Electrophoresis
Source: PLoS One. 2016 Dec 30;11(12):e0169259. doi: 10.1371/journal.pone.0169259 (PMC5201288; doi:10.1371/journal.pone.0169259)
Supplement: S3 Fig — (A) Western blot with antibody recognizing PCNA from fractions off the free flow electrophoresis. The fraction numbers are labeled on top of the corresponding lanes. (B) Coomassie stain of the blue native gel. (C) Western blot of fractions 37–61 from free flow electrophoresis fractions probed with antibody recognizing PCNA. (PDF) [file pone.0169259.s003.pdf]

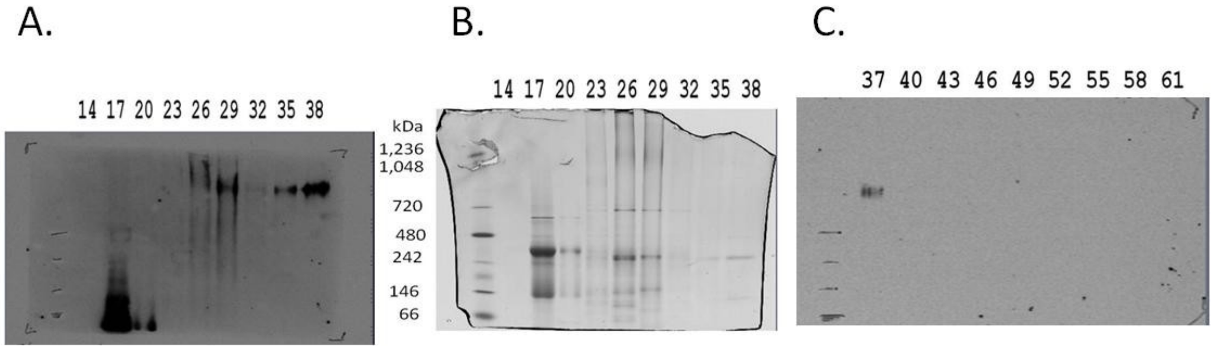

S3 Fig. Native molecular weight marker for Fig 3B. (A) Western blot with antibody recognizing PCNA from fractions off the free flow electrophoresis. The fraction numbers are labeled on top of the corresponding lanes. (B) Coomassie stain of the blue native gel. (C) Western blot of fractions 37-61 from free flow electrophoresis fractions probed with antibody recognizing PCNA.
